# The Control of Lay Women in Hospitals

**Published:** 1916-07-29

**Authors:** 


### The Control of Lay Women in Hospitals.

*To the Editor of* The Hospital.

Sir,—With regard to the question of women workers other than nurses being under the control of the matron of the hospital, one of the solutions is the courteous right feeling of the matron and of those employed—*i.e.*, of lay workers perhaps engaged by the Board without particular reference to the matron. The authority of a modern matron, except in the case of probationers, is greatly modified and more limited than in the days when education was less general and the woman's movement unborn. The work of women as dispensers, storekeepers, and clerks is outside the profession of a nurse, for which in particular the matron stands responsible.

But someone must be responsible to the Board of Management for the domestic arrangements, seemly conduct, and regularity of the lay women workers, or the lady dispensers, clerks, and storekeepers will soon find far more discomfort than they bargained for by "being under no matron." It would add to expense and confusion to appoint a new officer in charge of the lay staff, but no body of men or women, however small a battalion, can "work on their own," and a head there must be to secure comfort and efficiency. If the hospital matron, she should leave a wide margin to those lay women as to "time off" and all the little things, often made into petty stings. So long as they conform to the ordinary rules made for the good of all, which the matron herself must obey if she wants peace of mind, a certain liberty is needful and should be granted, although hospital regulations cannot be ignored, and can only be brought to notice by the responsible head. The matrons who remember that these lay helpers, never having had the benefit (?) of a three years' discipline, cannot easily understand the necessarily rigid routine of ward sisters and nurses, and that a welcome to dispenser, clerk, and storekeeper may introduce a new and pleasant element helpful to the matron and the nursing staff, should find no real difficulty in adding to their duties by the extra charge. It is questionable if the best type of woman ever resents reasonable authority. Experience teaches that a probationer who objects to authority is an unsuitable candidate for the nursing profession, and will make trouble if kept.—Yours faithfully,

Many Years a Matron.

